# Development of a deep learning model for early gastric cancer diagnosis using preoperative computed tomography images

**DOI:** 10.3389/fonc.2023.1265366

**Published:** 2023-10-06

**Authors:** Zhihong Gao, Zhuo Yu, Xiang Zhang, Chun Chen, Zhifang Pan, Xiaodong Chen, Weihong Lin, Jun Chen, Qichuan Zhuge, Xian Shen

**Affiliations:** ^1^ Zhejiang Engineering Research Center of Intelligent Medicine, The First Affiliated Hospital of Wenzhou Medical University, Wenzhou, China; ^2^ School of Information and Safety Engineering, Zhongnan University of Economics and Law, Wuhan, China; ^3^ Wenzhou Data Management and Development Group Co., Ltd., Wenzhou, Zhejiang, China; ^4^ School of Public Health and Management, Wenzhou Medical University, Wenzhou, Zhejiang, China; ^5^ Department of Gastrointestinal Surgery, The First Affiliated Hospital of Wenzhou Medical University, Wenzhou, China; ^6^ Zhejiang Provincial Key Laboratory of Aging and Neurological Disorder Research, Department of Neurosurgery, The First Affiliated Hospital of Wenzhou Medical University, Wenzhou, Zhejiang, China

**Keywords:** early gastric cancer (EGC), deep learning, CT, automatically stomach segmentation, gastric cancer classification

## Abstract

**Background:**

Gastric cancer is a highly prevalent and fatal disease. Accurate differentiation between early gastric cancer (EGC) and advanced gastric cancer (AGC) is essential for personalized treatment. Currently, the diagnostic accuracy of computerized tomography (CT) for gastric cancer staging is insufficient to meet clinical requirements. Many studies rely on manual marking of lesion areas, which is not suitable for clinical diagnosis.

**Methods:**

In this study, we retrospectively collected data from 341 patients with gastric cancer at the First Affiliated Hospital of Wenzhou Medical University. The dataset was randomly divided into a training set (n=273) and a validation set (n=68) using an 8:2 ratio. We developed a two-stage deep learning model that enables fully automated EGC screening based on CT images. In the first stage, an unsupervised domain adaptive segmentation model was employed to automatically segment the stomach on unlabeled portal phase CT images. Subsequently, based on the results of the stomach segmentation model, the image was cropped out of the stomach area and scaled to a uniform size, and then the EGC and AGC classification models were built based on these images. The segmentation accuracy of the model was evaluated using the dice index, while the classification performance was assessed using metrics such as the area under the curve (AUC) of the receiver operating characteristic (ROC), accuracy, sensitivity, specificity, and F1 score.

**Results:**

The segmentation model achieved an average dice accuracy of 0.94 on the hand-segmented validation set. On the training set, the EGC screening model demonstrated an AUC, accuracy, sensitivity, specificity, and F1 score of 0.98, 0.93, 0.92, 0.92, and 0.93, respectively. On the validation set, these metrics were 0.96, 0.92, 0.90, 0.89, and 0.93, respectively. After three rounds of data regrouping, the model consistently achieved an AUC above 0.9 on both the validation set and the validation set.

**Conclusion:**

The results of this study demonstrate that the proposed method can effectively screen for EGC in portal venous CT images. Furthermore, the model exhibits stability and holds promise for future clinical applications.

## Introduction

Gastric cancer (GC) is a highly prevalent malignancy, ranking among the top three in terms of mortality (1). Worldwide, over 1 million new cases of gastric cancer are diagnosed annually (2). The five-year survival rate for advanced gastric cancer (AGC) is less than 30%, while early gastric cancer (EGC) boasts a remarkable 90% survival rate (3, 4). EGC refers to invasive gastric cancer that penetrates no deeper than the submucosa, regardless of lymph node metastasis (5). The mainstay of treatment for EGC is endoscopic resection, while AGC is treated with sequential chemotherapy (5), with preoperative and adjuvant chemotherapy improving outcomes (6). Despite the vital implications for prognosis and treatment planning, the detection rate of EGC remains low, with even developed countries reporting a mere 50% diagnostic rate for EGC (3).

Gastric cancer is diagnosed histologically after endoscopic biopsy and staged using CT, endosonography(EUS), PET, and laparoscopy (5). The American Joint Committee on Cancer (8th edition) recommends computed tomography (CT) and endosonography as preoperative diagnostic techniques for gastric cancer. CT aids in identifying malignant lesions (7), detecting lymph node metastasis (8), and evaluating response to neoadjuvant chemotherapy (9). However, the highest reported accuracy for CT-based EGC detection is a mere 0.757 (10, 11), and the overall diagnostic accuracy for T staging is only 88.9% (12). Research indicates that EUS outperforms CT in preoperative T1 and N staging of gastric cancer (13, 14), yielding an overall T staging accuracy of 77% (15). Double contrast-enhanced ultrasonography (DCEUS) achieves a modest 82.3% accuracy in assessing gastric cancer T staging (16). Additionally, EUS staging is less effective in special locations such as the gastroesophageal junction (17).

The field of medical image analysis has witnessed significant interest in the application of rapidly advancing artificial intelligence techniques. These techniques have been successfully employed in various tasks such as image segmentation (18), disease detection (19), and lesion classification (20). Alam et al. utilized deep learning technology to automatically segment the gastrointestinal tract on MRI, aiding physicians in formulating precise treatment plans for cancer-affected regions of the gastrointestinal tract (21). Arai et al. developed a machine learning-based approach to accurately stratify the risk of gastric cancer, enabling individualized prediction of gastric cancer incidence (22). Ba et al., working with 110 whole slide images (WSI), compared deep learning with the diagnostic results of pathologists in diagnosing gastric cancer. The study demonstrated that deep learning technology indeed enhances the accuracy and efficiency of pathologists in gastric cancer diagnosis (23). Zeng et al. manually delineated gastric cancer lesions in portal phase CT images and subsequently selected the largest tumor slice and adjacent slices to establish a deep learning model for distinguishing between EGC and advanced gastric cancer (AGC) (24). However, manual delineation of lesion areas is time-consuming and demands a high level of expertise from the annotators, making it unsuitable for practical clinical diagnosis. This study aims to achieve automatic gastric segmentation and EGC detection on portal phase CT images by establishing a deep learning model capable of accurately distinguishing EGC.

## Materials and methods

### Patients

The study included 674 gastric cancer (GC) patients who underwent CT and pathological examinations at the First Affiliated Hospital of Wenzhou Medical University between January 2020 and April 2023. All patients were confirmed to have gastric cancer by pathological examination. Exclusion criteria consisted of the following: (1) patients who did not undergo enhanced CT scans, (2) patients with insufficient CT image quality, (3) patients with concurrent malignant tumors, and (4) patients who received neoadjuvant chemotherapy prior to CT examination. **Supplementary Figure 1** presents the flow chart outlining the inclusion and exclusion criteria, ultimately resulting in a final sample of 341 patients. Based on pathological examination results, all GC patients were categorized as either early gastric cancer (EGC, n=124) or advanced gastric cancer (AGC, n=217). They were randomly assigned to a training set (n=273) and a validation set (n=68) at an 8:2 ratio. Pathological examination findings served as the gold standard for gastric cancer staging. The Hospital Medical Ethics Committee approved this retrospective study.

### Image acquisition and preprocessing

Enhanced CT scans were performed using a TOSHIBA_MEC_CT3 device model. The scanning parameters were as follows: tube voltage range of 120 kVp, tube current range of 90-350 mA, table speed of 69.5 mm/rot, image matrix of 512×512, and reconstruction slice thickness of 2 mm. During the contrast-enhanced scan, 1.5 mL/kg of iodine contrast agent was injected through the antecubital vein using a syringe at a flow rate of 3.50 mL/s. Following the contrast medium injection, the patient held their breath, and imaging was conducted in the arterial phase (at 35-40s), portal venous phase (at 60-90s), and equilibrium phase (at 110s-130s).

Imaging during the portal venous phase is beneficial for assessing visceral invasion in surrounding tissues, as well as detecting and diagnosing lymph node metastasis, peritoneal metastasis, and extramural vascular invasion. Previous studies have employed this phase for tumor lesion segmentation (25, 26). To minimize the impact of exceptional cases on the model, we applied a window width of 350 Hounsfield units (Hu) and a window level of 50 Hu to truncate the grayscale values of CT images. Additionally, we normalized the grayscale values of all images to the range of [0,1].

### Two-stage deep learning model development

**Figure 1:**
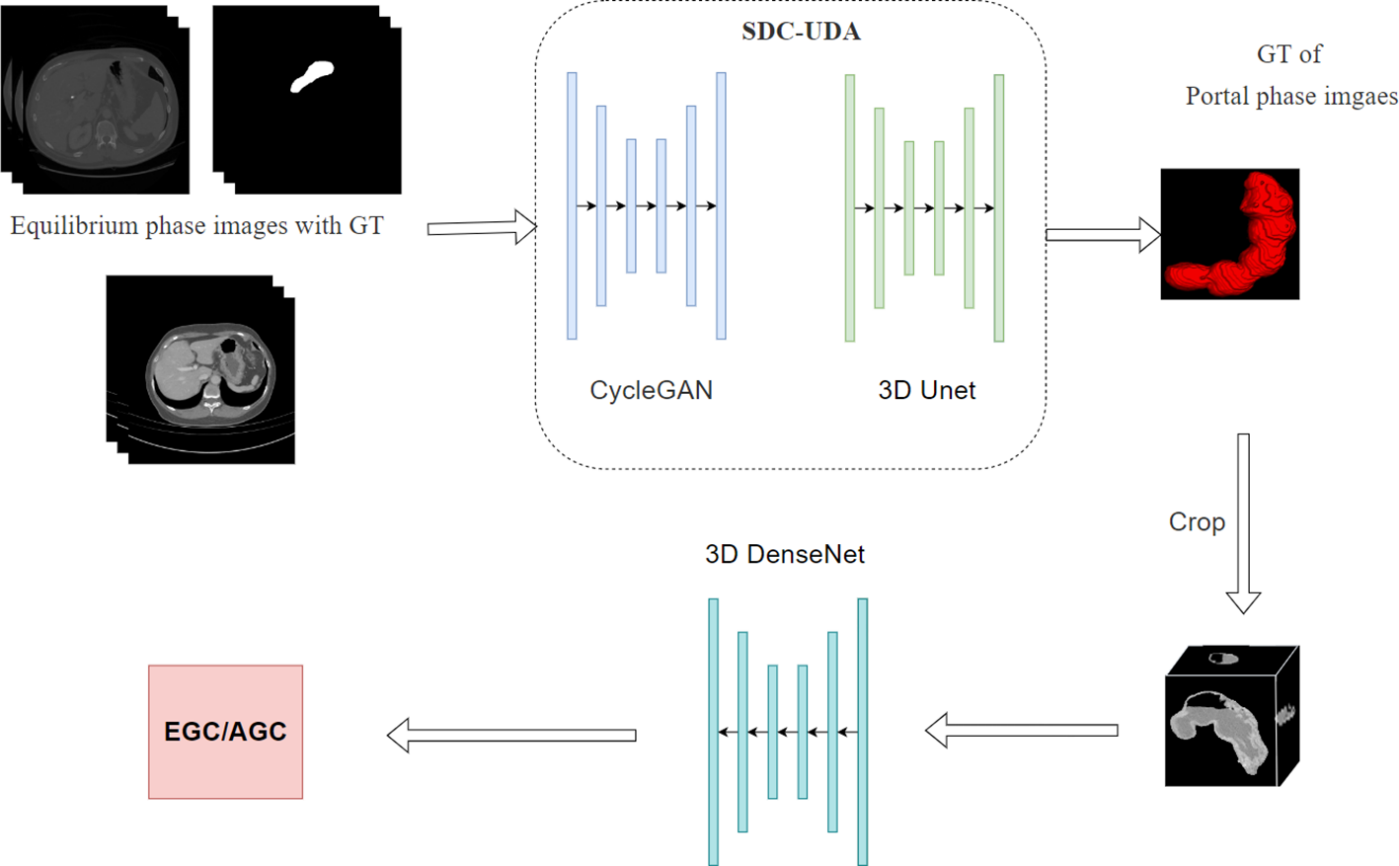
Structure of Deep Learning System Used for Stomach segmentation and EGC Diagnosis.

To facilitate fully automated distinction between early gastric cancer (EGC) and advanced gastric cancer (AGC), we have developed a two-stage deep learning model. As illustrated in **Figure 1**, the first stage of the model encompasses a two-dimensional segmentation network responsible for segmenting the stomach in enhanced CT images. The second stage involves a three-dimensional classification network dedicated to EGC screening.

### Segment model development

For the segmentation network in the first stage, we adopted the Slice-Direction Continuous Unsupervised Domain Adaptation Framework (SDC-UDA) (27). This framework leverages the equilibrium phase CT dataset with ground truth (GT) to achieve segmentation of the portal phase CT dataset without GT. The equilibrium phase CT dataset with GT was sourced from the MICCAI FLARE 2022 competition (https://flare22.grand-challenge.org/), comprising a total of 50 cases.

**Figure 2:**
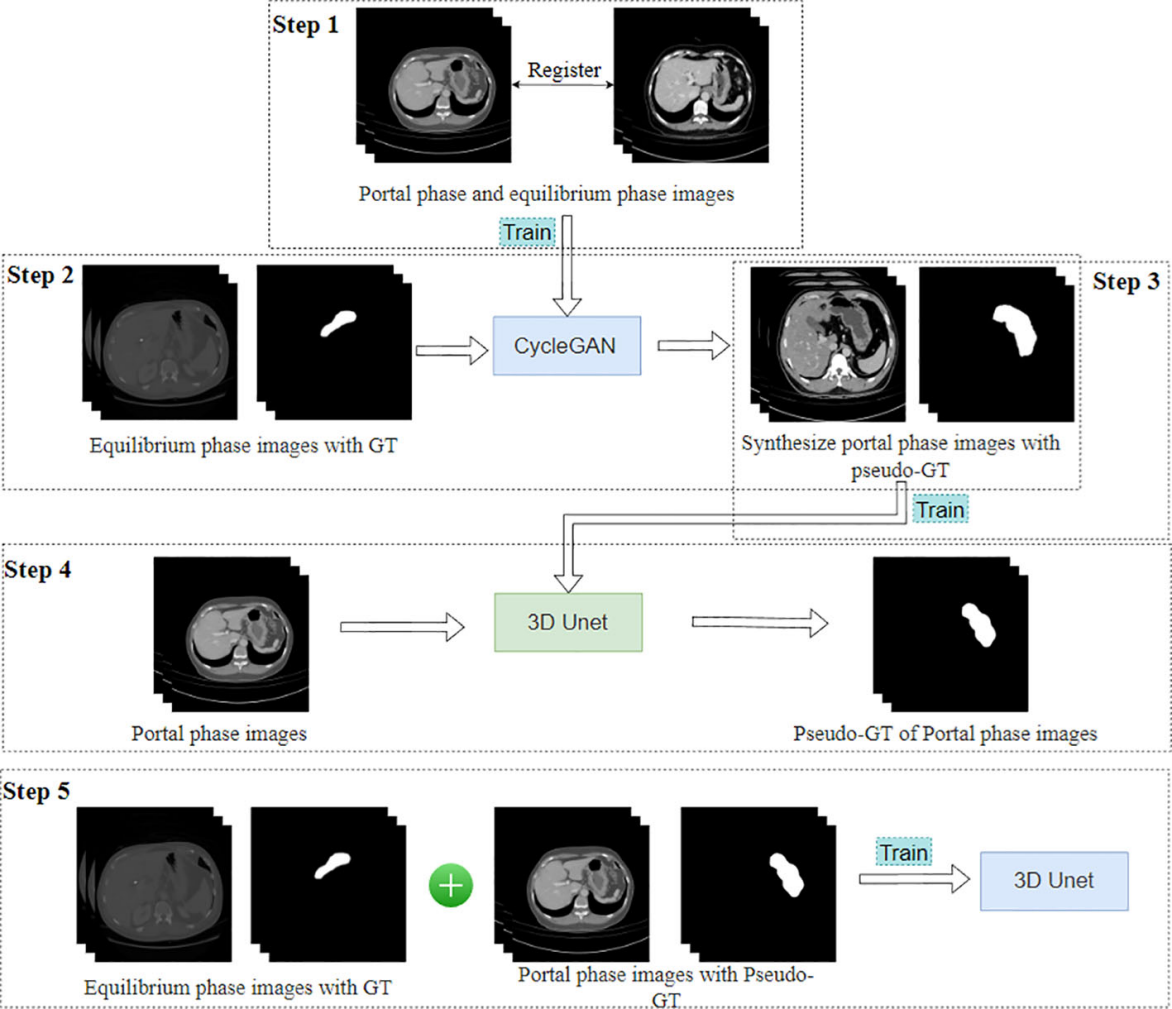
Overview of SDC-UDA framework.

The SDC-UDA model consists of five stages (**Figure 2**). Firstly, on our dataset, we registered the portal phase and equilibrium phase images and trained an unsupervised image translation generator, employing the CycleGAN network (28), with intra- and inter-slice self-attention. Secondly, we utilized the generator from the previous step to synthesize portal phase images with pseudo-GT, obtained from the equilibrium phase data with GT through 2D-to-3D image translation. Thirdly, we trained the synthesized portal phase images with pseudo-GT using the 3D-Unet network (29). Fourthly, we generated pseudo-GT for real portal phase images without GT using the 3D-Unet network trained in the previous step. Additionally, we improved the pseudo-GT through uncertainty-constrained pseudo-GT refinement. Finally, we jointly trained the segmentation model based on the 3D-Unet network by combining the equilibrium phase image with GT and the real portal phase image with the pseudo-GT.

In our hospital’s CT dataset, 50 cases of gastric segmentation were randomly selected by a doctor with 8 years of clinical experience using ITK-SNAP (version 3.8.0, USA). The segmentation results were utilized to evaluate the performance of the SDC-UDA model.

### Classification model development

**Figure 3:**
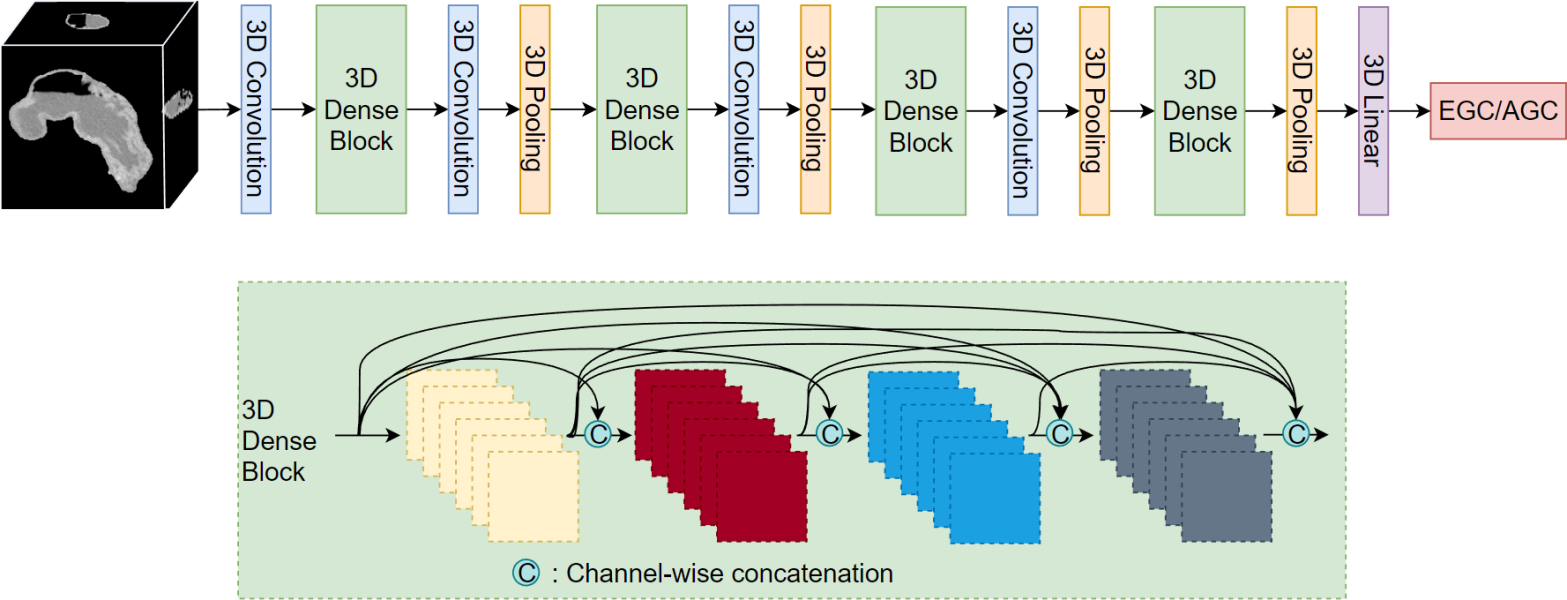
3D DenseNet network structure diagram.

After segmenting the stomach in the CT image, we extracted the stomach region based on the segmentation results and resized it to a dimension of 128*128*128. Subsequently, we employed the 3D DenseNet network (30) for the classification of EGC and AGC. The network comprises four dense modules (**Figure 3**), connected by convolutional and pooling layers. The final classification result is obtained through a linear layer after passing the output of the last dense module through the pooling layer. Each dense module consists of four convolutional blocks. The convolutional layer incorporates multiple convolutional layers, and the output of each convolutional block is concatenated with the outputs of all subsequent convolutional blocks in a channel-wise manner. Notably, all convolution kernels in the model are 3D.

### Model evaluation

To evaluate the segmentation model, we employed the dice coefficient (31). For the classification model, we assessed its performance by computing the accuracy, sensitivity, specificity, and F1 score. The receiver operating characteristic (ROC) curve was plotted, and the area under the ROC (AUC) value was calculated.

To assess the model’s stability, we randomly divided the data into three separate sets for training and validation, maintaining consistent proportions. We compared the results obtained from the three validation sets.

### Statistical analysis

All calculations and statistical analyses were conducted in a Linux environment (Ubuntu 7.5.0) using the following hardware configuration: an Intel 4215FR CPU clocked at 3.20 GHz, 64 GB DDR4 memory, and an RTX 4060 Ti graphics card. The programming language utilized was Python 3, specifically version 3.6.13 from the Python Software Foundation. We employed the PyTorch deep learning framework (https://pytorch.org/) along with key packages such as torch (version 1.10.1), torchvision (version 0.11.2), and scikit-learn (version 0.20.4).

## Results

### Patients

The study included a total of 341 cases of gastric cancer (GC). The training set comprised 273 participants (mean [SD] age: 66.02 [10.06] years), while the validation set consisted of 68 participants (mean [SD] age: 65.53 [10.62] years). Further details regarding the distribution of cases within the training and validation sets are presented in **Table 1**.

**Table 1:** The distribution of cases on the training and validation sets.

	Train set (n=273)	Validation set (n=68)	P-value
Age(years), (mean+SD)	66.02 ± 10.06	65.53 ± 10.62	0.832
Sex, n(%)			0.612
male	203(74.36)	50(73.53)	
female	70(25.64)	18(26.47)	
Gastric Cancer Staging, n(%)			0.357
EGC	97(35.53)	27(39.71)	
AGC	176(64.47)	41(60.29)	

### Model building

The optimal parameters for the model were determined through several experiments. For the CycleGAN in SDC-UDA, the parameters were set as follows: 100 training epochs, a beta value of 0.5, a learning rate of 0.0001, and learning rate updates every 50 epochs. The parameters for the 3D Unet in SDC-UDA were set as follows: 200 training epochs, a batch size of 2, a learning rate of 0.0005, and learning rate updates every 100 epochs. The parameters for the 3D DenseNet were set as follows: the base network was DenseNet121, a dropout rate of 0.5, a growth rate of 4, 1000 training epochs, a batch size of 20, a learning rate of 0.1, and learning rate updates every 500 epochs.

### Model performance evaluation

**Figure 4:**
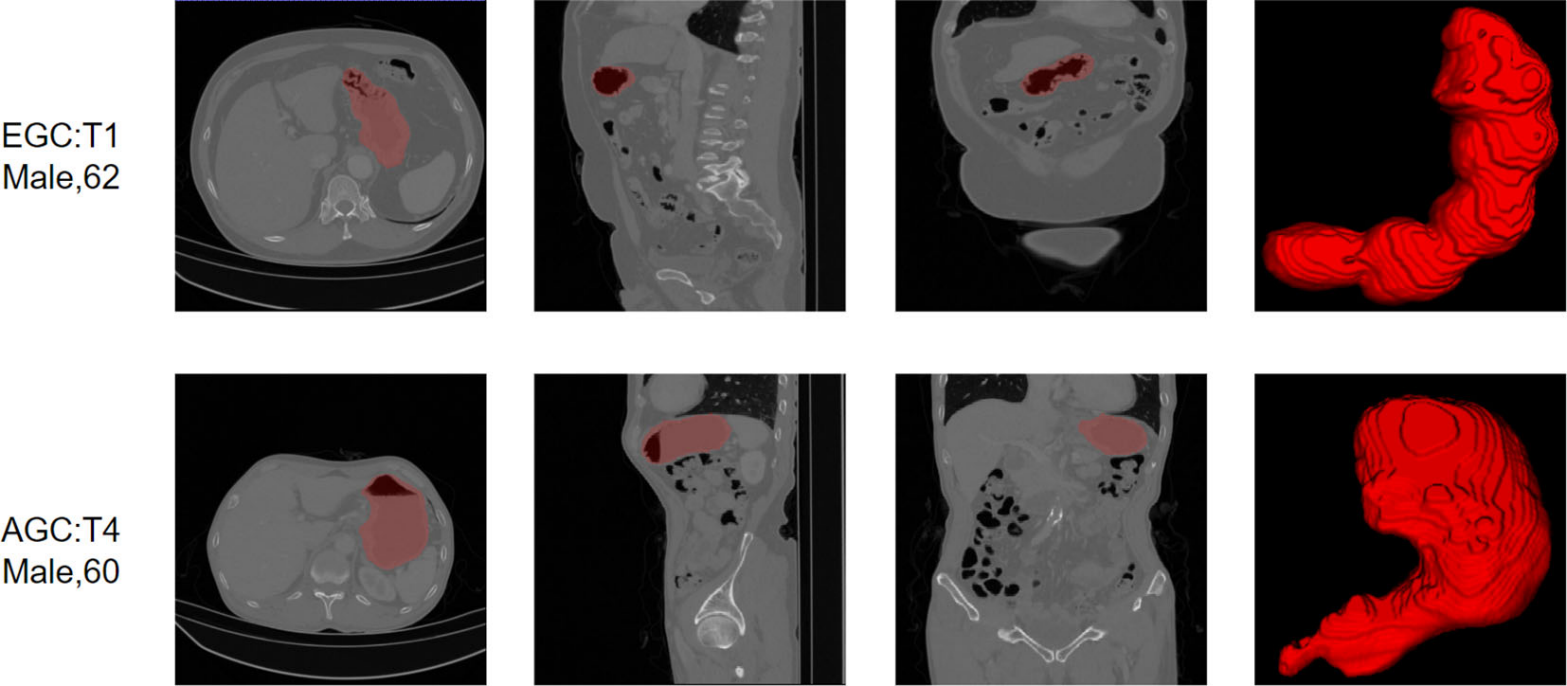
Two cases to show the segmentation of our model.

On a dataset of 50 human-annotated gastric segmentations, our model achieved an average dice accuracy of 0.94, with the highest dice coefficient recorded as 0.97 and the lowest as 0.90. The segmentation results are illustrated in **Figure 4**, demonstrating a close match between the model’s segmentation output and the actual stomach outline.

**Figure 5:**
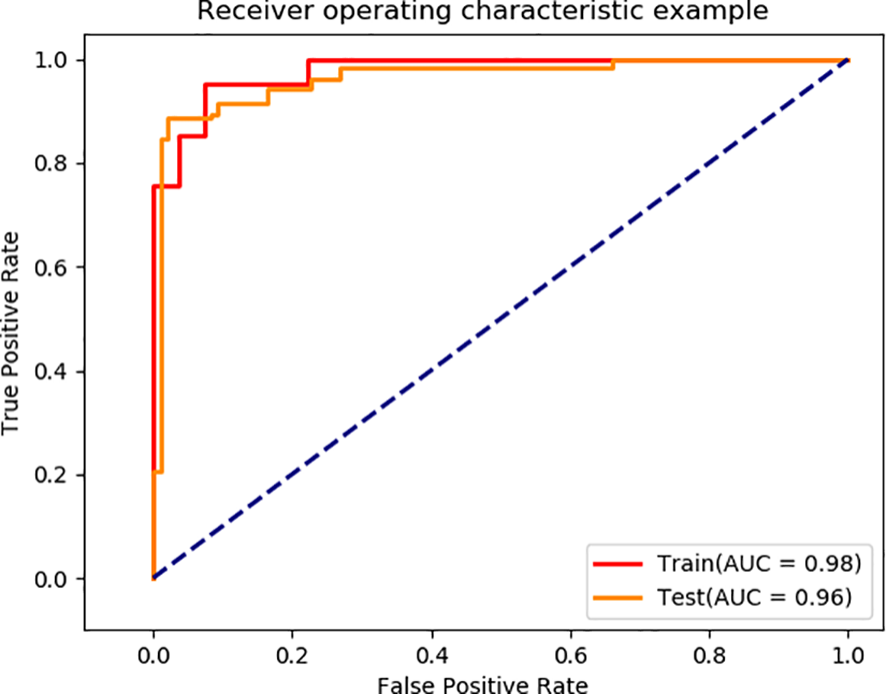
EGC screening performance of our model.

Using the segmented images as input for EGC detection, the final model achieved AUC values of 0.98 and 0.96 on the training and validation sets, respectively (**Figure 5**). The model’s performance metrics on the training set are as follows: accuracy of 0.93, sensitivity of 0.92, specificity of 0.92, and F1 score of 0.93 (**Table 2**). On the validation set, the model achieved an accuracy of 0.92, sensitivity of 0.90, specificity of 0.89, and F1 score of 0.93. These experimental results demonstrate that the proposed model exhibits high discriminative ability in distinguishing between EGC and AGC.

**Table 2:** The performance of our model on the train set and validation set.

	Train Set					Validation set				
	AUC	ACC	SEN	SPE	F1	AUC	ACC	SEN	SPE	F1
Our mode	0.98	0.93	0.92	0.92	0.93	0.96	0.92	0.90	0.89	0.93

ACC, accuracy; SEN, sensitivity; SPE, specificity.

### Model robustness assessment

To examine the impact of different data distributions on the model, we randomly divided the data into training and validation sets in an 8:2 ratio, performed model training and testing, and repeated this process more than three times. The robustness of the model was evaluated based on the performance of the three models. **Supplementary Figure 2** and **Supplementary Table 1** present the model’s performance on various training and validation sets. The results indicate that the model achieved an AUC greater than 0.90 on both the training set and validation set, demonstrating the robustness of the deep learning model proposed in this paper.

## Discussion

In this study, we developed a deep learning model for accurate EGC screening using CT images without human annotation. The model consists of two stages: automatic gastric segmentation and EGC diagnosis. It achieved AUC values of 0.98 and 0.96 in the training set and validation set, respectively. Additionally, our model demonstrated robustness in EGC screening through multiple training iterations with varied data groupings. Our study provides a clinically applicable preoperative GC staging model for the GC patients, which can assist doctors in formulating more accurate diagnosis and treatment plans.

Preoperative diagnosis of GC has been a focal point of research. Accurately assessing the T stage in the GC classification system is crucial for determining treatment options and prognosis. Understaging may lead to incomplete tumor resection, while overstaging may result in unnecessary overtreatment. EUS could be helpful for identifying superficial that do not penetrate further than the submucosa (T1) or muscularis propria (T2) from advanced cancers (T3–T4) (5). Studies have reported that EUS achieves a high overall accuracy in T stage assessment, with a sensitivity of 86% (32). Zhao et al. demonstrated that the accuracy of staging using multi-slice spiral CT images and gastroscope can reach 83.67% through statistical analysis (33). Guan et al. employed Yolov5-based DetectionNet for staging gastric cancer on arterial phase CT images, achieving an average accuracy of 0.909 (34). Wang et al., using gastric windows on CT images, achieved an accuracy rate of 90% in diagnosing T1 EGC (35). In our study, the model achieved an accuracy of 94.6% in diagnosing EGC in the training set and 90% in the internal validation set. Furthermore, the reliability study demonstrated the stability of the model.

Artificial intelligence technologies, such as radiomics and deep learning, have gained significant attention in the field of gastric cancer. These techniques play a crucial role in tasks like preoperative TNM staging prediction, differential diagnosis, treatment response assessment, and prognosis estimation (36). Radiomics, in particular, has been employed for predicting treatment response and survival in gastric cancer, although there is heterogeneity and relatively low research quality in this area. Nonetheless, radiomics holds promise in predicting clinical outcomes due to its high interpretability (37). An essential initial step in radiomics is the segmentation of the region of interest (ROI), which can be time-consuming and demanding for annotators (38, 39). Thus, automatic segmentation of the ROI is of great significance for omics research. Hu Z et al. proposed a multi-task deep learning framework for automatic segmentation of gastric cancer in human tissue sections using whole slide images (WSI) (40). Zhang Y et al. presented a 3D multi-attention-guided multi-task learning network for gastric tumor segmentation on CT images, achieving a Dice score of 62.7% by leveraging complementary information from different dimensions, scales, and tasks (41). However, there are limited studies on the segmentation of gastric cancer CT, and the Dice accuracy falls short of meeting the requirements of subsequent experiments. Therefore, the model we developed focuses on achieving automatic segmentation of the stomach in the first stage.

This paper used a two-stage deep learning framework that first segments and then classifies. This framework has a wide range of applications in the medical field. In brain disease research, many researchers first remove the skull by segmentation, and then perform subsequent classification modeling (42, 43). In order to classify 18 types of brain tumors more accurately, Gao et al. (44) adopted a deep learning framework that first segmented the tumor area and then performed multi-classification. For the diagnosis of chest diseases, researchers often use segmentation models to extract lung areas, and then perform nodule detection (45), Covid-19 detection (46), interstitial lung disease (47), etc. Compared with models that directly use original images for classification, the two-stage deep learning framework can first segment the area where the target of interest is located, so that the classification model focuses on the target area, which not only improves efficiency, but also improves classification accuracy.

Despite the promising potential of AI in the field of gastric cancer, its clinical application has been hindered by its low interpretability (48, 49). In our study, we utilized the Gradient Weighted Class Activation Map (Grad-CAM) technique to visualize the regions of focus in the model. However, this technique only provided information about the areas the model concentrated on, without revealing the specific features on which the model relied to classify EGC versus AGC (**Supplementary Figure 3**). Therefore, it is essential to conduct further analysis of the model’s interpretability and verify its reliability from a clinical perspective.

This study has several limitations: Firstly, it is a retrospective study, which may introduce statistical biases. Subsequent studies will include more prospective investigations. Secondly, all the data used in this study originated from a single center. Therefore, future research should incorporate data from multiple centers to develop a more general and robust system. Thirdly, the patients included in this study were exclusively those with pathologically diagnosed gastric cancer. Consequently, the EGC detection model proposed in this paper may not be suitable for detecting EGC in CT images without gastric cancer. Lastly, this study solely focused on CT images in the portal phase, and subsequent research will explore joint multi-sequence CT image modeling.

## Conclusion

We have developed a deep learning model that automates the screening of EGC in CT images of patients with GC. The model follows a three-step process: first, it performs stomach segmentation; then, it crops the segmented stomach region; and finally, it feeds the cropped images into the classification network for EGC screening. As the entire process is computer-based, this model holds significant clinical value by assisting doctors in assessing the gastric cancer status of patients and devising personalized treatment plans.

## Data availability statement

The raw data supporting the conclusions of this article will be made available by the authors, without undue reservation.

## Ethics statement

The studies involving humans were approved by The Ethics Review Board of the First Affiliated Hospital of Wenzhou Medical University and Wenzhou Central Hospital. The studies were conducted in accordance with the local legislation and institutional requirements. The participants provided their written informed consent to participate in this study.

## Author contributions

ZG: Conceptualization, Formal Analysis, Funding acquisition, Investigation, Project administration, Writing – original draft. ZY: Data curation, Methodology, Writing – original draft. XZ: Software, Writing – original draft. CC: Writing – original draft. ZP: Writing – original draft. XC: Writing – original draft. WL: Writing – original draft. JC: Writing – original draft. QZ: Writing – review & editing. XS: Writing – review & editing.
