# Supplementary material for: Development of a deep learning model for early gastric cancer diagnosis using preoperative computed tomography images
Source: Front Oncol. 2023 Oct 6;13:1265366. doi: 10.3389/fonc.2023.1265366 (PMC10587601; doi:10.3389/fonc.2023.1265366)
Supplement: Supplementary file 1 [file DataSheet_1.docx]

**Supplementary Figure 1.**
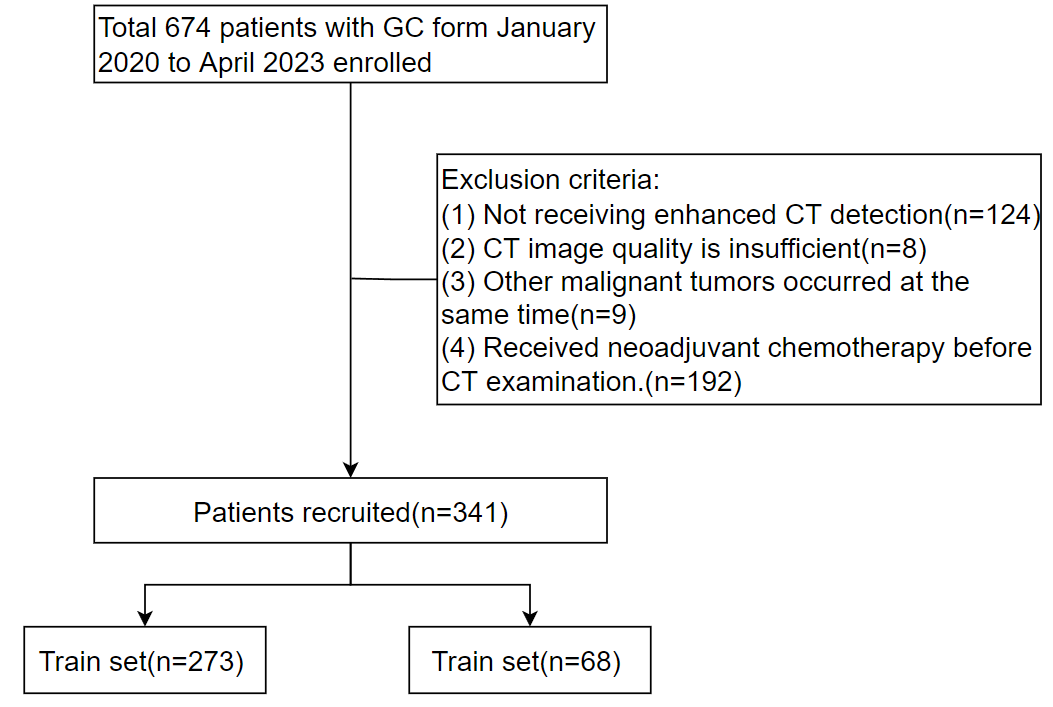
Flow chart of patients recruitment in this study.


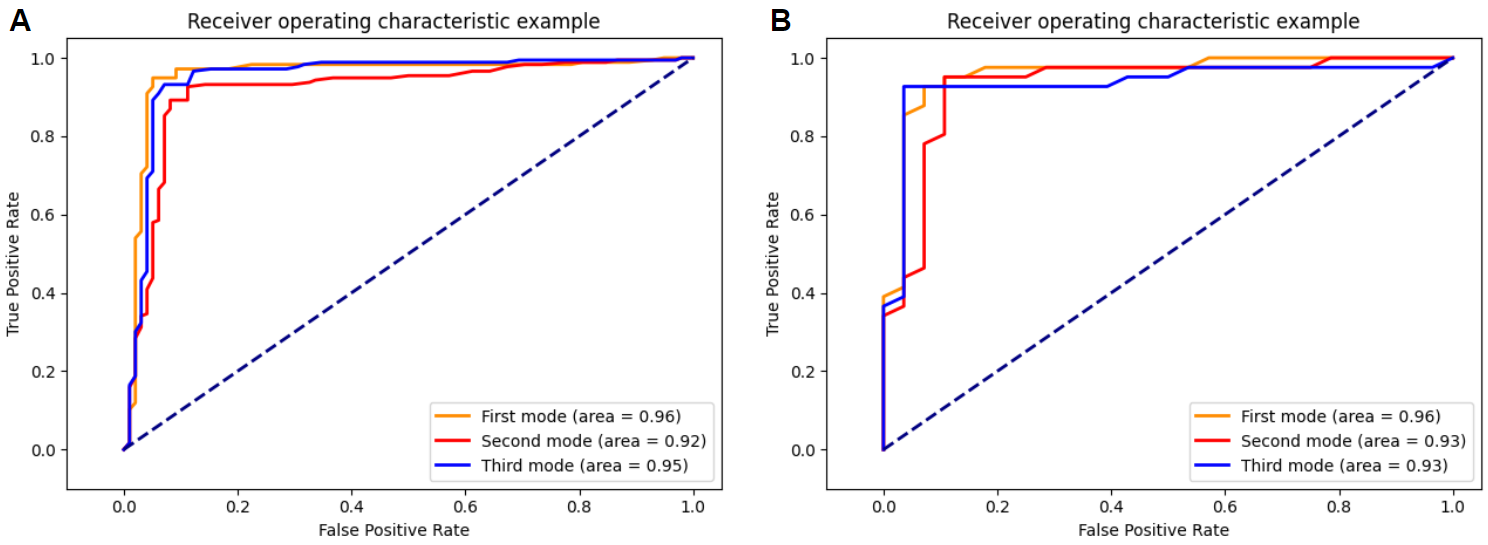


**Supplementary Figure 2.** The ROCs of the models on the training set and test set after three different data groupings. A)ROCs of train set. B) ROCs of test set.


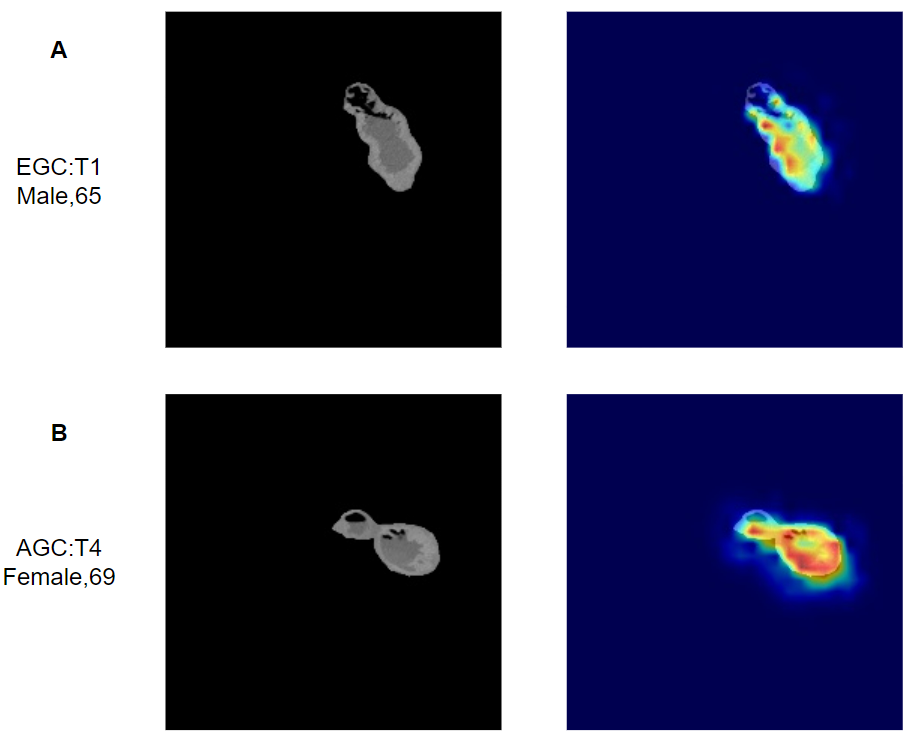


**Supplementary Figure 3.** Activation maps for deep learning models. A)Tumor invasion: limited to the superficial layer of the mucosa(T1). B)Tumor invasion: mostly invades subserosal connective tissue but not serosa, small areas invade serosa (T4a)

**Supplementary Table 1**. The performance of the models on the train set and test set after three different data groupings.

|  | Train Set | | | | |  | Test Set | | | | |
| --- | --- | --- | --- | --- | --- | --- | --- | --- | --- | --- | --- |
|  | AUC | ACC | SEN | SPE | F1 |  | AUC | ACC | SEN | SPE | F1 |
| First mode | 0.96 | 0.93 | 0.91 | 0.95 | 0.91 |  | 0.96 | 0.91 | 0.89 | 0.93 | 0.90 |
| Second mode | 0.92 | 0.89 | 0.89 | 0.89 | 0.85 |  | 0.93 | 0.93 | 0.86 | 0.91 | 0.89 |
| Third mode | 0.95 | 0.92 | 0.89 | 0.93 | 0.88 |  | 0.93 | 0.94 | 0.96 | 0.93 | 0.93 |

ACC:accuracy; SEN:sensitivity; SPE:specificity.
